# Arbuscular mycorrhizal fungi enhance soil nutrient cycling by regulating soil bacterial community structures in mango orchards with different soil fertility rates

**DOI:** 10.3389/fmicb.2025.1615694

**Published:** 2025-06-27

**Authors:** Yuling Han, Guoyin Yuan, Xiaolong Yang, Longfa Fang, Yu Liang, Baoyuan Zhou, Zhiyuan Wei

**Affiliations:** ^1^Tropical Crop Genetic Resources Institute, Chinese Academy of Tropical Agricultural Sciences, Haikou, China; ^2^National Key Laboratory for Tropical Crop Breeding, Sanya, China; ^3^Key Laboratory of Crop Gene Resources and Germplasm Enhancement in Southern China, Ministry of Agriculture and Rual Affairs, Haikou, China; ^4^Key Laboratory of Tropical Crops Germplasm Resources Genetic Improvement and Innovation of Hainan Province, Haikou, China; ^5^College of Ecology, Lishui University, Lishui, China; ^6^Hubei Key Laboratory of Food Crop Germplasm and Genetic Improvement, Institute of Food Crops, Hubei Academy of Agricultural Sciences, Wuhan, China; ^7^State Key Laboratory of Herbage Improvement and Grassland Agro-Ecosystems, College of Pastoral Agriculture Science and Technology, Lanzhou University, Lanzhou, China; ^8^Key Laboratory of Crop Physiology and Ecology, Institute of Crop Sciences, Chinese Academy of Agricultural Sciences, Ministry of Agriculture and Rural Affairs, Beijing, China

**Keywords:** arbuscular mycorrhizal fungi (AMF), soil bacterial community, soil properties, soil enzymes, soil metabolites, fruit tree

## Abstract

Arbuscular mycorrhizal fungi (AMF) substantially enhance soil fertility and are widely recognized as one of the most extensively researched biological inoculants. However, the effects of AMF on soil nutrient availability in mango orchards, along with the physiological processes regulating this availability under varying soil fertility conditions, remain poorly understood. To address this research gap, experiments were conducted with two soil types (soil from mango orchards co-cultivated with butterfly pea plants for 5 years) and a control (no butterfly pea plants) and two mycorrhizal inoculations (+AMF and –AMF). *Rhizophagus irregularis* was used as the mycorrhizal inoculum. These experiments examined the physicochemical properties, microbial community composition, and metabolic profiles in mango orchard soils by integrating high-throughput sequencing with soil metabolomics. In nutrient-poor soils, the introduction of AMF enhanced the occurrence of specific bacterial species and elevated the richness and diversity of the soil bacterial community. This enhancement subsequently increased the activities of soil enzymes such as cellulase, *β*-glucosidase, chitinase, and nitrate reductase in mycorrhizosphere soil, leading to improved soil pH, and increases in soil microbial biomass carbon (MBC), ammonium nitrogen (NH_4_^+^-N), nitrate nitrogen (NO_3_^−^-N), available potassium (K), and available phosphorus (P). Furthermore, alterations in soil properties and metabolites restructured the soil microbial community, with pH and MBC emerging as the key factors shaping bacterial distribution within mycorrhizosphere soil co-occurrence networks. In contrast, the effects of AMF on soil nutrient availability were weaker in high-fertility soils. We conclude that AMF enhance soil nutrient availability in mango orchards by regulating soil bacterial community structures, particularly in low-fertility soils.

## 1 Introduction

Arbuscular mycorrhizal fungi (AMF) establish symbiotic associations with approximately 80% of land plants (Jiang et al., 2020). These fungi facilitate the exchange of mineral nutrients, particularly nitrogen (N) and phosphorus (P), for carbon provided by plants during symbiosis (Liu et al., 2022; Gou et al., 2023). This nutrient exchange is mediated by specific membrane transporters located in root cortex cells, which form newly established host-microbe interfaces known as arbuscules (Gutjahr and Parnisk, 2013). In previous studies, AMF have obtained 4–20% of the total photosynthetically fixed carbon from their hosts plant through a symbiotic relationship (Hodge, 1996). Carbon is supplied in the form of fatty acids and hexoses, which fuel the metabolic activity of the fungi and are converted into an extensive network of extraradical hyphae (Rich, 2017). AMF are crucial as a natural provider of nutrients for crops such as mangoes, apples, citrus, maize, and soybeans (Jiang et al., 2020; Aghili et al., 2014; Smith et al., 2011; Wu et al., 2017).

AMF can alter soil nutrient balance through modulating the composition of microbial communities (Yang et al., 2020). Several studies have shown that, after colonizing plant roots, AMF significantly change the mycorrhizosphere’s bacterial communities (Garbaye, 1994; Redecker et al., 2013). Tisserant et al. (2013) found that AMF have a notably weak exoenzymatic repertoire compared to other mycorrhizal fungi (e.g., ecto-, orchid, and ericoid) and saprotrophic fungi. However, Hodge et al. (2001) demonstrated that AMF can simultaneously promote the breakdown of complex organic matter in soil and enhance N uptake using dual-labeled (^15^N/^13^N) *Lolium perenne* leaves. Previous studies reveal that the mineralization of organic N in soil is facilitated by the interactions between AMF and other saprotrophic microorganisms (Thirkell et al., 2016). Toljander et al. (2007) reported that AMF can stimulate microbial growth by releasing labile substrates through exudation and hyphal turnover, thus promoting the growth of free-living microbial communities (Toljander et al., 2007). The findings of Zhang et al. (2016, 2019) indicate that AMF possess a restricted ability to mineralize organic P. However, these fungi can increase the capacity of P-solubilizing bacteria to generate enzymes that release P. Jiang et al. (2021) suggested that AMF hyphae act as a “transport network” for bacteria, facilitating their efficient access to patches of organic N and P. This transport enhances the utilization of these discrete nutrient sources.

AMF colonization can affect root metabolic activities, resulting in alterations to root exudates and the assembly of unique mycorrhizosphere microbial communities (Marschner and Crowley, 1997). The soil microbiota is essential for nutrient cycling, breaking down organic matter, and storing carbon (Coban et al., 2022). Soil microorganisms produce extracellular enzymes like cellulase, chitinase, *β*-glucosidase, and nitrate reductase, which influence the mineralization of carbon and N (Lioussanne et al., 2010). Soil enzymes serve as sensitive indicators of changes in soil quality, showing strong correlations with biological activity in soils. These enzymes help directly mediate the biological breakdown of both organic and mineral components in soil (Brooks et al., 2013; Balota et al., 2004). To date, most studies have examined the interactions between microbes and plant roots. However, less attention has been paid to the relationships between AMF and their associated microbial communities, as well as the impact of these interactions on nutrient uptake efficiency through the mycorrhizal pathway.

Orchards cover a substantial area of land worldwide and are traditional agroecosystems (Rey, 2011). Orchard ecosystems are more stable and semipermanent than short-term cropping systems, such as maize fields (Han et al., 2021). In Hainan Province, China, mango orchards have formed a leading industry due to their multiple advantages. Mango trees rely heavily on AMF symbiosis to obtain the necessary water and nutrients that are crucial for their growth and fruit development (Augé, 2001). Oehl et al. (2010) and Baltruschat et al. (2019) reported that plants may have varying degrees of dependence on AMF for nutrient assimilation from soils with different fertility levels. Furthermore, AMF in China have been suggested as effective bioinoculants for organic farming (Wang et al., 2016). Previous research demonstrated negative correlations between AMF and soil available nutrients (including nitrate nitrogen (NO_3_^−^-N), ammonium nitrogen (NH_4_^+^-N), and available P) in mango orchards, indicating the need for comprehensive consideration of soil nutrients when fertilizing in AMF-associated orchard systems (Jiang et al., 2020). However, there remains a knowledge gap regarding how AMF influence soil nutrients under both high and low fertility conditions, particularly through their interactions with soil bacterial communities in regulating nutrient dynamics. Although AMF associations represent a crucial component for maintaining agricultural ecosystem sustainability, their specific impacts on mango orchard systems require further investigation. Although AMF associations are integral components in maintaining agroecosystem sustainability, the impacts of these fungi on mango orchards require further investigation. Thus, we sought to assess the impact of AMF in high-and low-nutrient soils on available soil nutrients, root exudates, soil microorganisms, and the interrelationships among these factors. Additionally, the study aimed to examine quantitative AMF fertilizer management strategies under different soil fertility levels.

## 2 Materials and methods

### 2.1 Experimental design

The experiment utilized *Rhizophagus irregularis* (Justo et al., 2010) as the mycorrhizal inoculum, which was supplied by the Plant Nutrition and Resources Institute of the Beijing Academy of Agriculture and Forestry Sciences. Prior to the experiment, the AMF were propagated for 4 months in a greenhouse using maize (*Zea mays* L.) as the host plant. The culture medium comprised a mixed matrix of zeolite and sand in a 1:3 ratio. The mixture was subjected to sterilization in an autoclave, where it was maintained at 0.14 MPa pressure and 121°C temperature for 2 h. To maintain soil moisture, sterile water was applied to the maize seedlings every 3 d, and Hoagland nutrient solution was added on a weekly basis. After 4 months of maize growth, the mycorrhizal inoculum was collected as a mixture of root fragments, sand, hyphae, and AMF spores, with the spore density exceeding 25 spores/g.

Surface (0–40 cm) soil samples were collected from a mango orchard (18°19′54.1´´N, 109°24′52.7″E) in Sanya, Hainan Province, China, which is located in a tropical marine climate. A field experiment was conducted in the mango orchard starting in 2017, with two treatments: (H) butterfly pea (*Clitoria ternatea*) planting and (C) a no-butterfly pea control. The experiment was conducted for 6 years until 2023, and planting butterfly pea substantially improved the available nutrients in the mango orchard’s soil. We collected surface soil samples (0–40 cm) from five sampling points arranged in a crisscross pattern within the mango cultivation areas of H and C. Soil H exhibited significantly higher fertility compared to C (Table 1). To eliminate large stones and plant debris, the collected soil samples were sieved using a 2 mm mesh, then thoroughly mixed and stored at room temperature. After sieving, the soil was immediately collected to determine the concentrations of NH_4_^+^ and NO_3_^−^. The soils were then dried naturally to measure pH, available P, and available potassium (K).

**Table 1:** Basic chemical and biological indicators for soil in mango orchards planted with butterfly pea and a control.

Soil	NO_3_^—^N mg/kg	Alkali-hydro N mg/kg	NH_4_^+^-N mg/kg	Available P mg/kg	Available K mg/kg	Chao index	Shannon index
H	15.41 a	45.53 a	1.26 a	5.46 a	216.00 a	16,768 b	4.96 a
C	5.75 b	17.66 b	1.09 a	3.81 a	75.8 b	19,227 a	4.95 a

Different lower-case letters in the same column indicate significant differences in different treatments (*p* < 0.05) (*n* = 6). NO_3_^−^-N, nitrate nitrogen; Alkali-hydro N, alkali-hydrolyzale nitrogen; NH_4_^+^-N, ammonium nitrogen; Available P, available phosphorus; Available K, available potassium; H, Mango orchard soil planted for 6 years with butterfly pea plants. C, Mango orchard soil with no butterfly pea planted.

The experiment included four treatments: two soil types (mango orchard soil planted with butterfly pea plants for 5 years and a control) and two mycorrhizal inoculations (+AMF or -AMF). Therefore, the experiment included four treatments: (1) AH, AM inoculation with planting in butterfly pea soil; (2) H, planting in butterfly pea soil without AM inoculation; (3) AC, AM inoculation in control soil and (4) C – control soil without AM inoculation. The number of replicates of each treatment was six. Three-compartment microcosm pots, consisting of the roots and fungal hyphae compartment (RHC), buffer compartment (BC) and hyphal compartment (HC) (Supplementary Figure S1). The pots had diameters of 26 cm (top) and 21 cm (bottom) and a height of 28 cm. The root hyphal compartment (RHC) was physically segregated by a nested PVC assembly comprising two vertically aligned cylindrical sections. The inner containment structure consisted of a PVC column (20 cm height × 10 cm internal diameter), concentrically nested within a larger-diameter outer casing (12 cm diameter) to create the buffer compartment (BC) between the RHC and the surrounding hyphal chamber (HC). A porous barrier system with 30 μm nylon mesh filters lined both cylindrical interfaces. This configuration confined root development to the RHC while permitting selective bidirectional hyphal transfer across compartments. The BC was designed primarily to mitigate nutrient flux in the soil. The mango cultivar used in this study was “Guifei” (*Mangifera indica* L. “Guifei”). Potted experiments were conducted using 50-day-old mango seedlings. AMF inoculation was performed via the layered inoculation method in root compartments were administered 50 g of inoculum, whereas those without AMF treatments were given sterilized inoculum 50 g. The experiment was carried out in a greenhouse at Danzhou (109°58′E, 19°52′N), which experiences a tropical monsoon climate with mean annual temperatures ranging from 17.5 to 33°C. Since the temperature in Danzhou was suitable for mango seedling growth, no heating or cooling measures were applied in the greenhouse. The greenhouse was ventilated on both sides, and its primary purpose was to shield the seedlings from rain. Approximately every 3 to 4 d, deionized water was added to maintain the soil moisture at 60% of its water-holding capacity. Each pot was weighed every 3 to 4 d to maintain soil water content. Additionally, mango saplings were watered monthly with 100 mL of Hoagland nutrient solution throughout the growth period.

### 2.2 Soil sampling and analysis

Three months after planting the mango saplings, the bulk soil was carefully removed from their root systems. The soil attached to the roots was categorized as mycorrhizosphere soil. Impurities were carefully removed using tweezers, and the rhizospheric soil was immediately placed in resealable bags. Soil samples from the bulk region were also collected from the HC. Samples were initially sieved through a 2 mm screen and then divided into three separate parts. A sample was air-dried to evaluate available P and soil enzyme activity under natural conditions. NO_3_^−^-N, NH_4_^+^-N, pH, and microbial biomass carbon (MBC) were analyzed using another sample stored at 4°C. For the microbe and metabolite analyses, the remaining samples were stored at −80°C.

A glass electrode (Mettler-Toledo FE20, Switzerland) was used to measure the soil pH in a 2.5:1 soil-to-water mixture (Wolińska et al., 2017). The concentrations of soil mineral N, including NH_4_^+^-N and NO_3_^−^-N, were measured using a colorimetric method with a continuous-flow analysis system (FIAstar 5,000, Foss Tecator AB, Sweden). The chloroform fumigation-extraction method was employed to determine the concentration of MBC (Collins et al., 2016). Using flame atomic absorption spectrometry, the concentration of available K in the soil was analyzed after extraction with 1 mol L^−1^ NH₄OAc. Soil available P was estimated using the molybdenum-antimony resistance method.

The collected soil samples were immediately placed in sample trays, spread into thin layers, and air-dried in a clean, well-ventilated indoor environment maintained at 28°C to avoid direct sunlight exposure. After drying, the samples were ground and sieved through 40-and 60-mesh sieves for soil enzyme analysis. Soil enzyme activities were determined using commercial assay kits manufactured by Nanjing Jiancheng Bioengineering Institute (China). A brownish-red substance was produced using 3,5-dinitrosalicylic acid and soil cellulase to catalyze the degradation of reducing sugars. Nitrate reductase catalyzed the reduction of nitrate to nitrite, which then reacted with color reagents to form red diazonium compounds under acidic conditions. *β*-glucosidase catalyzed the hydrolysis of the substrate to produce yellow p-nitrophenol. Chitinase hydrolyzed chitin oligomers to produce N-acetylglucosamine, which then reacted with ferrocyanide. The activities of cellulase, nitrate reductase, β-glucosidase, and chitinase were measured at 540, 540, 405, and 420 nm, respectively.

### 2.3 DNA extraction and amplicon sequencing

A Mag-Bind^®^ Soil DNA Isolation Kit (Omega Bio-Tek, Norcross, GA, United States) was used to extract DNA from 0.25 g of soil samples. A TBS-380 fluorometer (Promega, United States) was used to determine the DNA concentration, and a NanoDrop 2000 spectrophotometer (Thermo Fisher Scientific, United States) was employed to assess DNA purity. To evaluate the quality of the extracted DNA, 1% agarose gel electrophoresis was performed to examine its integrity.

Using the extracted DNA as template, bacterial 16S rRNA gene V3–V4 variable regions were amplified by PCR with barcode-carrying primers 338F (5’-ACTCCTACGGGAGGCAGCAG-3′) and 806R (5’-GGACTACHVGGGTWTCTAAT-3′). Fungal ITS regions were amplified using primers ITS1F (5’-CTTGGTCATTTAGAGGAAGTAA-3′) and ITS2R (5’-GCTGCGTTCTTCATCGATGC-3′). Sequencing was performed on the Illumina PE300/PE250 platform, and raw data were deposited in the NCBI SRA database. Quality-filtered sequences were clustered into operational taxonomic units (OTUs) at 97% similarity threshold using UPARSE v7.1, with chimera removal. Taxonomic annotation of OTUs was performed using the RDP classifier against the Silva 16S and ITS rRNA gene databases with a confidence threshold of 70%, followed by community composition analysis at different taxonomic levels.

### 2.4 Metabolite extraction and amplicon analysis

For sample preparation, a 50 mg soil sample was loaded into a 2 mL centrifuge tube equipped with a 6 mm grinding bead. To extract metabolites, a solution was prepared using a 4:1 volume ratio of methanol to water and supplemented with 0.02 mg/mL of the internal standard L-2-chlorophenylalanine. A volume of 400 μL of this solution was employed. A Wonbio-96c cryogenic tissue grinder from Shanghai Wanbo Biotechnology Co., Ltd. (China) was utilized to homogenize the sample. The homogenization was carried out at −10°C, with the grinder set to 50 Hz, and run for 6 min. Following the completion of the grinding, the sample was exposed to low-temperature ultrasonic extraction at 40 kHz and 5°C for 30 min, and subsequently incubated at −20°C for a further 30 min. Following centrifugation at 13,000 g for 15 min at 4°C, the supernatant was carefully moved to an injection vial for further LC–MS/MS analysis. The LC–MS analysis was performed using a SCIEX UPLC-TripleTOF system (ultra-high performance liquid chromatography-time-of-flight mass spectrometry) provided by Shanghai Majorbio Bio-pharm Technology Co., Ltd. After data acquisition, raw LC–MS data were processed using Progenesis QI software (Waters Corporation, United States) for baseline filtering, peak identification, integration, retention time correction, and alignment to generate a data matrix of retention times, mass-to-charge ratios (m/z), and peak intensities. Subsequently, both MS and MS/MS spectra were matched against public metabolite databases (HMDB and Metlin) and Majorbio’s in-house database for metabolite identification.

### 2.5 Statistical analyses

Before conducting ANOVA (with each indicator analyzed independently), dataset normality and variance homogeneity were verified using Shapiro–Wilk (*p* > 0.05) and Levene’s tests (*p* > 0.05). Non-normally distributed data underwent log transformation. The Tukey’s HSD test was used to identify significant differences (*p* < 0.05) between treatments in both baseline and post-experiment soil analyses Two-way ANOVA was used to measure the effects of arbuscular mycorrhizae (AM), soil type (Soil) and their interaction on soil nutrient availability and soil enzymes. Residuals were checked for a normal distribution using the Shapiro–Wilk test. The data were analyzed using the SPSS software, version 21.0 (SPSS Institute, Inc., Cary, NC, United States), and the figures were created with Origin 8.0. Microbial alpha diversity (Chao 1, Shannon, and Simpson indices) was assessed using mothur, and Wilcoxon rank-sum tests were employed to compare diversity between groups. Chao 1 was used to identify community richness, while the Shannon and Simpson were used to identify community diversity. A principal coordinate analysis (PCoA) based on the Bray–Curtis dissimilarity was conducted with the Vegan package (version 2.5–3) in R to examine the similarities of microbial communities across samples. To investigate how soil physicochemical properties affected bacterial community structures, a redundancy analysis (RDA) was performed using the same package. Statistically significant correlations between nodes were determined when Spearman’s correlation coefficients exceeded 0.5 or were below −0.5, with *p*-values less than 0.01. Bubble plots illustrating these correlations were created using the MetaboAnalyst software. An orthogonal partial least squares discriminant analysis (PLS-DA) was utilized, and variables with VIP (variable importance in projection) values greater than 1 were considered significant. A co-occurrence network of soil components was built based on normalized metabolic profiles, incorporating microbial genera and physicochemical properties.

## 3 Results

### 3.1 Effects of different treatments on the soil physicochemical characters

**Figure 1:**
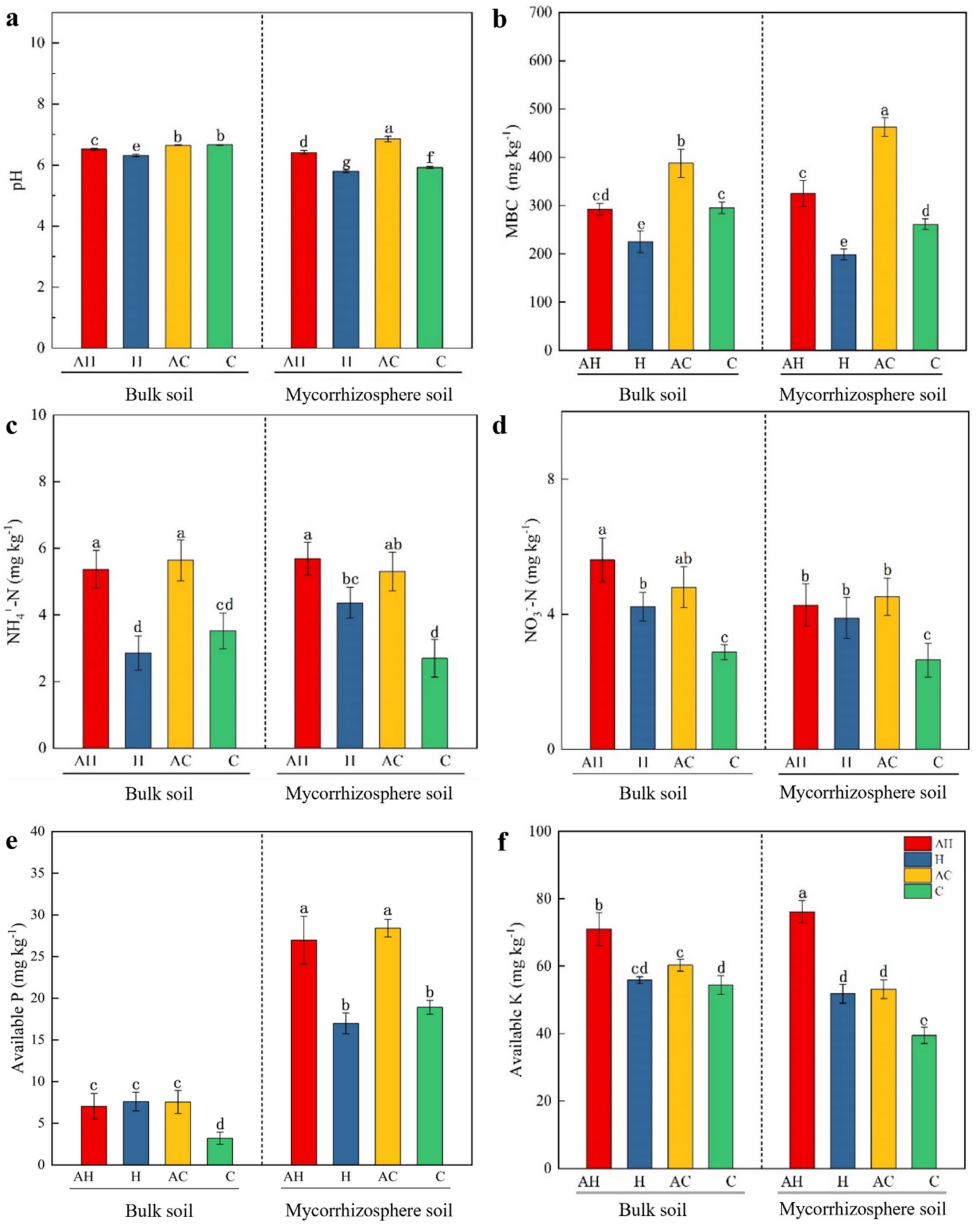
Effects of AM and butterfly pea on soil bulk and mycorrhizosphere properties: **(a)** pH, **(b)** MBC, **(c)** NH_4_^+^-N, **(d)** NO_3_^−^-N, **(e)** Available P, and **(f)** Available K contents. pH, soil pH; MBC, microbial biomass carbon; NH_4_^+^-N, ammonium nitrogen; NO_3_^−^-N, nitrate nitrogen; Available P, available phosphorus; Available K, available potassium. AH, AM with planting in butterfly pea soil; H, planting in butterfly pea soil; AC, AM with control soil (no planting butterfly pea soil); and C, control soil (no planting butterfly pea soil). Different lower letters indicating significant differences among these treatments. Error bars indicate ± standard errors (*n* = 6).

In the AH treatment, the bulk soil pH was significantly greater (3.1%) than that observed in the H treatment (Table 2; Figure 1a). In comparison to the C treatment, the mycorrhizosphere soil pH in the AH and AC treatments significantly increased (8.3 and 15.7%, respectively). In the AC treatment, the MBC content in the bulk soil was significantly greater (31.3%) than in the C treatment (Figure 1b). A significantly higher MBC content was observed in the mycorrhizosphere soil of the AH treatment (24.4%) and the AC treatment (77.1%) relative to the C treatment. Relative to the C treatment, the NH_4_^+^-N concentrations in the bulk soil were significantly higher in the AH (52.8%) and AC treatments (60.4%) (Figure 1c). In the mycorrhizosphere soil, the NH_4_^+^-N concentrations significantly increased in the AH (111.1%) and AC (96.5%) treatments, relative to the C treatment. Relative to the C treatment, the NO_3_^−^-N concentrations in the bulk soil of the AH (95.1%) and AC (66.8%) treatments were significantly higher (Figure 1d). The NO_3_^−^-N concentrations in the mycorrhizosphere soil showed significant increases of 61.2% in the AH treatment and 70.5% in the AC treatment, relative to the C treatment. Compared to the bulk soil, the concentration of available P in the mycorrhizosphere soil was significantly increased for the AH, H, AC, and C treatments (Figure 1e). In the mycorrhizosphere soil, the available P concentration was significantly elevated by 42.6% in the AH treatment and by 50.2% in the AC treatment, relative to the C treatment. In the bulk soil, the available K concentration was significantly elevated by 30.4% in the AH treatment and by 10.8% in the AC treatment, relative to the C treatment (Figure 1f). In the mycorrhizosphere soil, the available K concentration was significantly elevated by 93.1% in the AH treatment and by 34.8% in the AC treatment, relative to the C treatment.

**Table 2:** Significance due to the effect of arbuscular mycorrhizae (AM), planting in butterfly pea soil or no planting soil (Soil) and their interaction (AM × Soil) on the soil nutrient availability from the data in Figure 1, using Two-way ANOVA.

Source	df	pH	MBC	NH_4_^+^-N	NO_3_^−^-N	Available P	Available K
AM	1	17.61^**^	95.33^**^	58.39^**^	29.81^**^	2.39	42.85^**^
Soil	1	6.54^*^	53.35^**^	1.02	9.62^**^	0.02	27.59^**^
AM × Soil	1	0.08	4.02	0.62	3.96	0.04	4.88^*^

df, degree of freedom; pH, soil pH; MBC, microbial biomass carbon; NH_4_^+^-N, ammonium nitrogen; NO_3_^−^-N, nitrate nitrogen; Available P, available phosphorus; Available K, available potassium. Significant levels: ^**^ *p* < 0.01, ^*^ *p* < 0.05.

### 3.2 Changes of cellulase, nitrate reductase, *β*-glucosidase, and chitinase activities in different treatments

**Figure 2:**
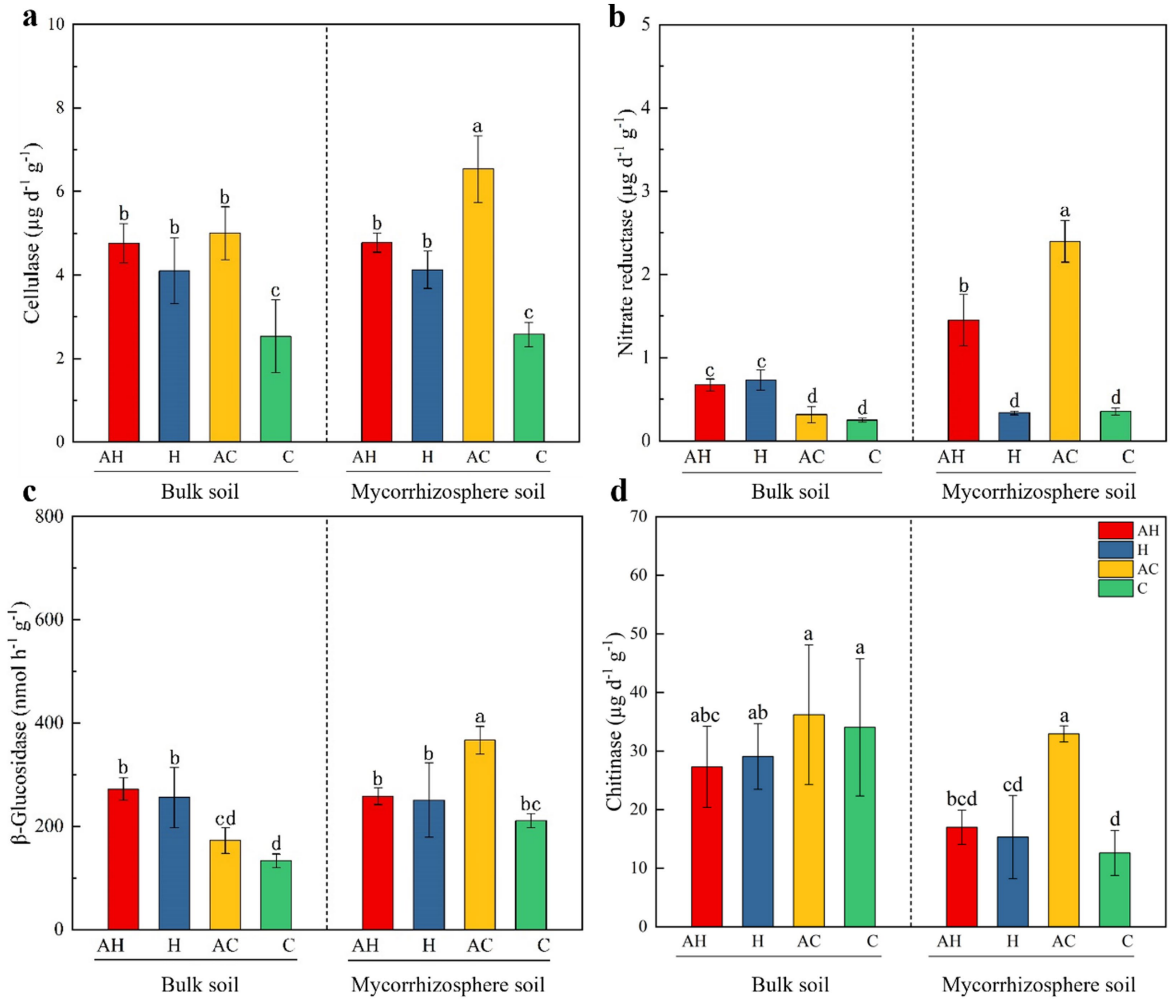
Effects of AM and butterfly pea on bulk and mycorrhizosphere soil **(a)** cellulase, **(b)** nitrate reductase, **(c)** *β*-glucosidase, and **(d)** chitinase. Cellulase, nitrate reductase, β-glucosidase and chitinase indicate soil cellulase, nitrate reductase, β-glucosidase and chitinase activities, respectively. AH, AMF in butterfly pea planting soil; H, butterfly pea planting soil; AC, AMF with control soil (no planting butterfly pea soil); C, control soil (no planting butterfly pea soil). Different lower letters indicating significant differences among these treatments. Error bars indicate ± standard errors (*n* = 6).

The various treatments had distinct effects on enzyme activity (Table 3; Figure 2). In the mycorrhizosphere soil, the AC treatment exhibited significantly higher activities of cellulase, nitrate reductase, β-glucosidase, and chitinase compared to the other treatments (Figure 2). Compared to the C treatment, the cellulase activity in the bulk soil increased significantly by 87.9, 61.7, and 97.2% in the AH, H, and AC treatments, respectively. In the mycorrhizosphere soil, these increases were 85.1, 60.0, and 153.6% for the AH, H, and AC treatments, respectively (Figure 2a). Relative to the C treatment, the nitrate reductase activity in the mycorrhizosphere soil of the AH (309.5%) and AC (104.5%) treatments were significantly higher (Figure 2b). In the mycorrhizosphere soil, the AC treatment significantly increased β-glucosidase activity by 73.8% and chitinase activity by 161.6%, compared to the C treatment. In contrast, the AC treatment had no significant impact on enzyme activities in the bulk soil when compared to the C treatment (Figures 2c,d).

**Table 3:** Significance due to the effect of arbuscular mycorrhizae (AM), planting in butterfly pea soil or no planting soil (Soil) and their interaction (AM × Soil) on the soil enzymes availability from the data in Figure 1, using Two-way ANOVA.

Treatment	df	Cellulase	Nitrate reductase	β-glucosidase	Chitinase
AM	1	47.08^**^	7.76^*^	2.09	1.86
Soil	1	0.97	1.06	0.02	2.73
AM × Soil	1	20.57^**^	2.44	1.54	1.89

df, degree of freedom; Cellulase, nitrate reductase, β-glucosidase and chitinase indicate soil cellulase, nitrate reductase, β-glucosidase and chitinase activities, respectively. Significant levels: ^**^ *p* < 0.01, ^*^ *p* < 0.05.

### 3.3 Soil bacterial diversity

In both the bulk and mycorrhizosphere soils, the treatments showed significant differences in bacterial diversity metrics, including the Chao1, Shannon, and Simpson indices (Table 4). Relative to the C treatment, the AC treatment demonstrated significantly higher values for the Chao1, Shannon, and Simpson indices in the mycorrhizosphere soil. Relative to the C treatment, the Chao1 and Simpson indices in the AH and H treatments remained non-significantly different. Furthermore, in the bulk soil, the AC and C treatments exhibited no significant variations in the Chao1, Shannon, and Simpson indices.

**Table 4:** Changes in bacteria diversity in the bulk and mycorrhizosphere soil under different treatments.

Soil	Treatments	Chao1	Shannon	Simpson
Bulk	AH	20077.67 ± 649.55 b	4.83 ± 0.07 a	0.037 ± 0.002 b
	H	19839.00 ± 222.19 b	4.86 ± 0.02 a	0.038 ± 0.000 b
	AC	22032.33 ± 243.38 a	4.94 ± 0.08 a	0.042 ± 0.002 a
	C	21883.00 ± 171.08 a	4.86 ± 0.05 a	0.039 ± 0.002 ab
Mycorrhizosphere	AH	18172.33 ± 71.84 c	5.04 ± 0.02 a	0.036 ± 0.002 b
	H	18192.00 ± 245.05 bc	4.90 ± 0.04 b	0.034 ± 0.006 b
	AC	19955.00 ± 679.78 a	5.11 ± 0.08 a	0.052 ± 0.003 a
	C	18898.00 ± 250.96 b	4.71 ± 0.05 c	0.035 ± 0.002 b

AH, AMF in butterfly pea planting soil; H, butterfly pea planting soil; AC, AMF with control soil (no planting butterfly pea soil); C, control soil (no planting butterfly pea soil). Data are presented as means ± SE (*n* = 3), where different letters within columns denote significant differences (*p* < 0.05).

### 3.4 Bacterial and fungal community composition in the bulk and mycorrhizosphere soil

**Figure 3:**
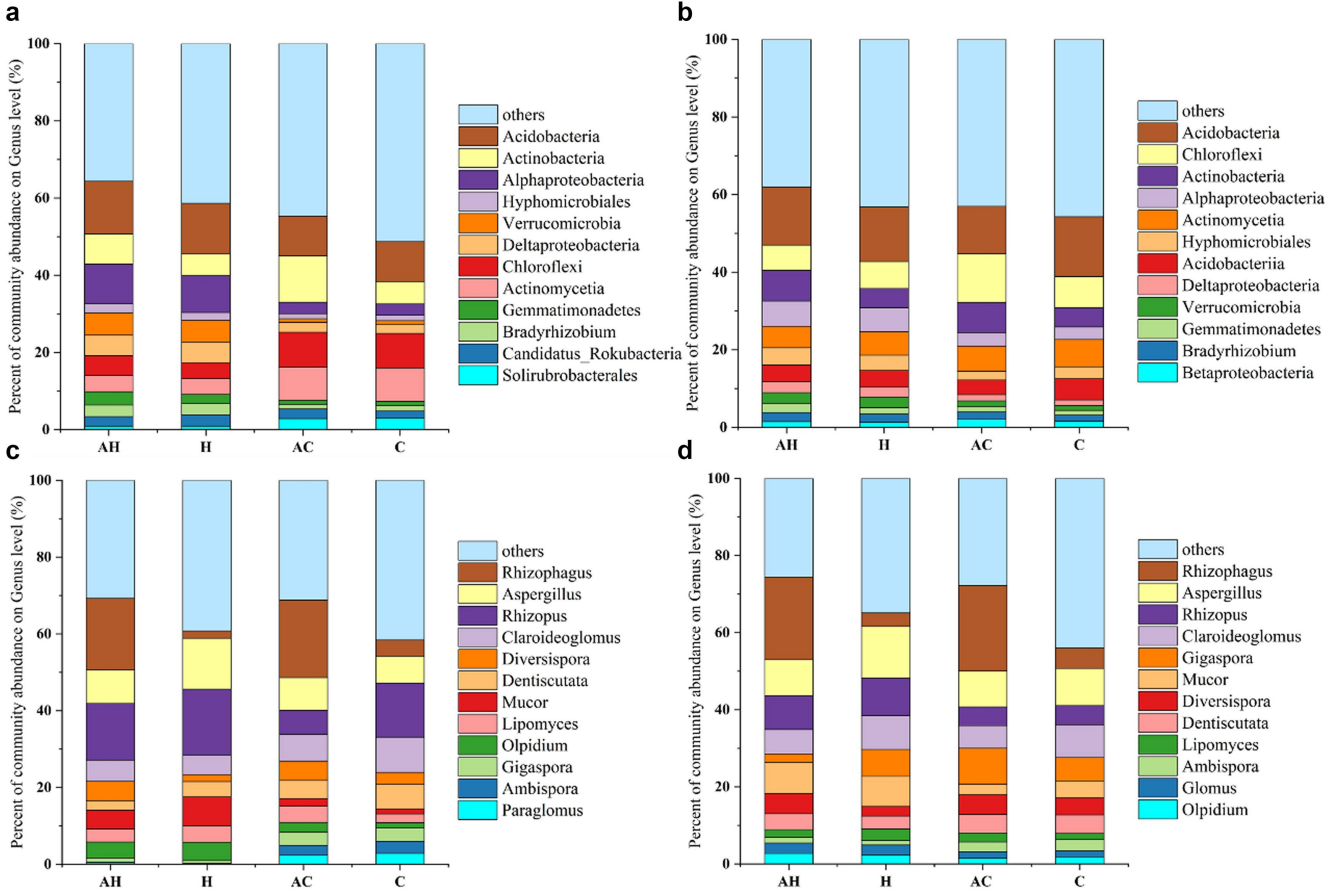
Relative abundances of bacteria in the **(a)** bulk and **(b)** mycorrhizosphere soils, and fungi in the **(c)** bulk and **(d)** mycorrhizosphere soils under different treatments (*n* = 3). AH, AMF in butterfly pea planting soil; H, butterfly pea planting soil; AC, AMF with control soil (no planting butterfly pea soil); C, control soil (no planting butterfly pea soil).

In the bulk soil of all treatments, the dominant genera included unclassified members of _p_*Acidobacteria*, _c_*Alphaproteobacteria*, _o_*Hyphomicrobiales*, _p_*Verrucomicrobia*, _c_*Deltaproteobacteria*, _p_*Chloroflexi*, and _c_*Actinomycetia* (Figure 3a). In the mycorrhizosphere soil of AH treatments, the most dominant genera were unclassified members of _p_*Acidobacteria*, _p_*Chloroflexi*, _c_*Alphaproteobacteria*, _c_*Actinomycetia*, and _o_*Hyphomicrobiales* (Figure 3b).

Across both the bulk and mycorrhizosphere soils of all treatments, the most abundant fungal genera were *Rhizophagus*, *Diversispora*, *Aspergillus*, *Rhizopus*, and *Claroideoglomus* (Figures 3c,d). AMF significantly increased the relative abundances of *Rhizophagus* and *Diversispora* in both bulk and mycorrhizosphere soils (*p < 0.05*). In contrast, the relative abundances of *Claroideoglomus*, *Dentiscutata*, *Gigaspora*, *Ambispora*, and *Paraglomus* showed no significant differences between the AMF and butterfly pea treatments.

**Figure 4:**
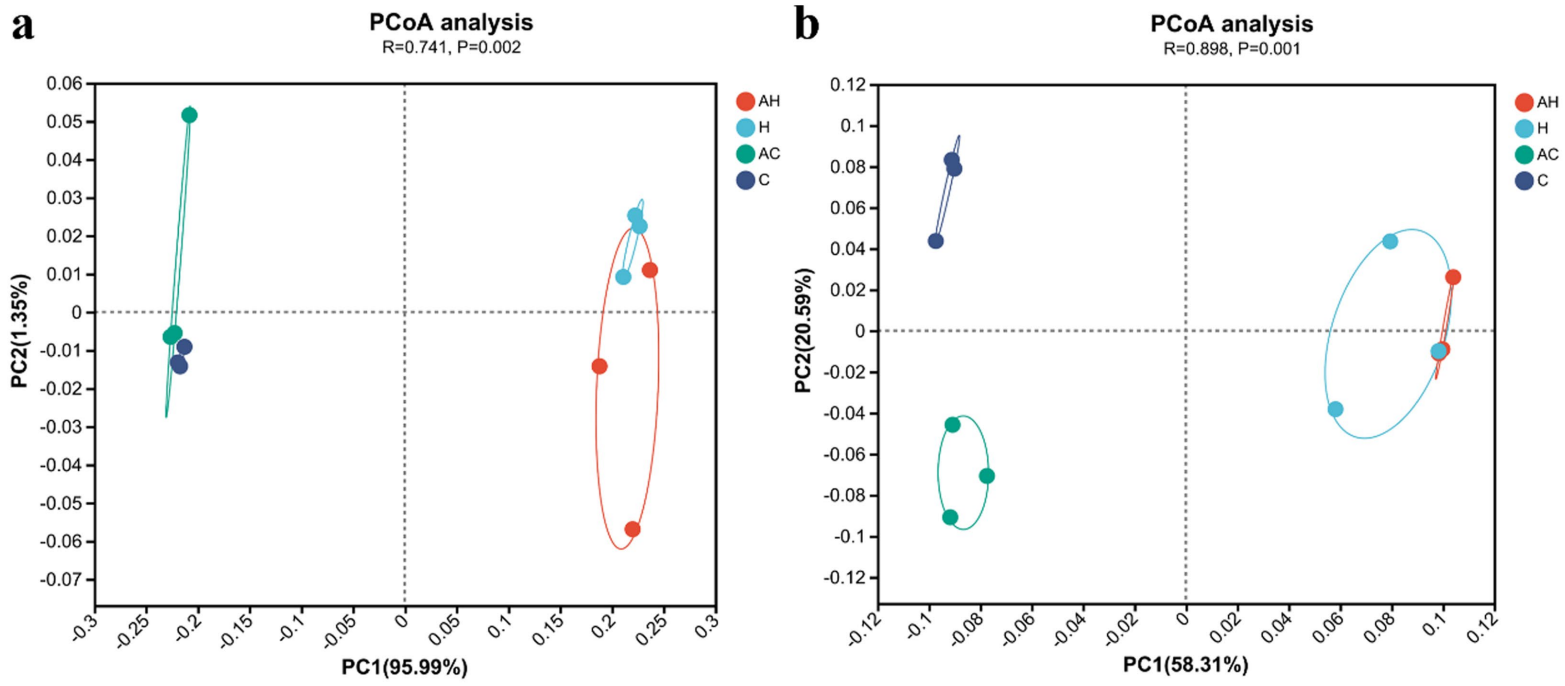
Principal coordinate analysis of the **(a)** bulk and **(b)** mycorrhizosphere soils bacterial communities under different treatments. AH, AMF in butterfly pea planting soil; H, butterfly pea planting soil; AC, AMF inoculated control soil; C, control soil without AMF inoculation.

At the genus level, PCoA based on the distance matrix separated each group in the bulk (Figure 4a) and mycorrhizosphere (Figure 4b) soils. The first principal component (PC1, 95.99%) separated the AH and H soil samples from the AC and C soil samples in the bulk soil, and the samples showed the same trend in the mycorrhizosphere soil with the first principal component (PC1, 58.31%).

**Figure 5:**
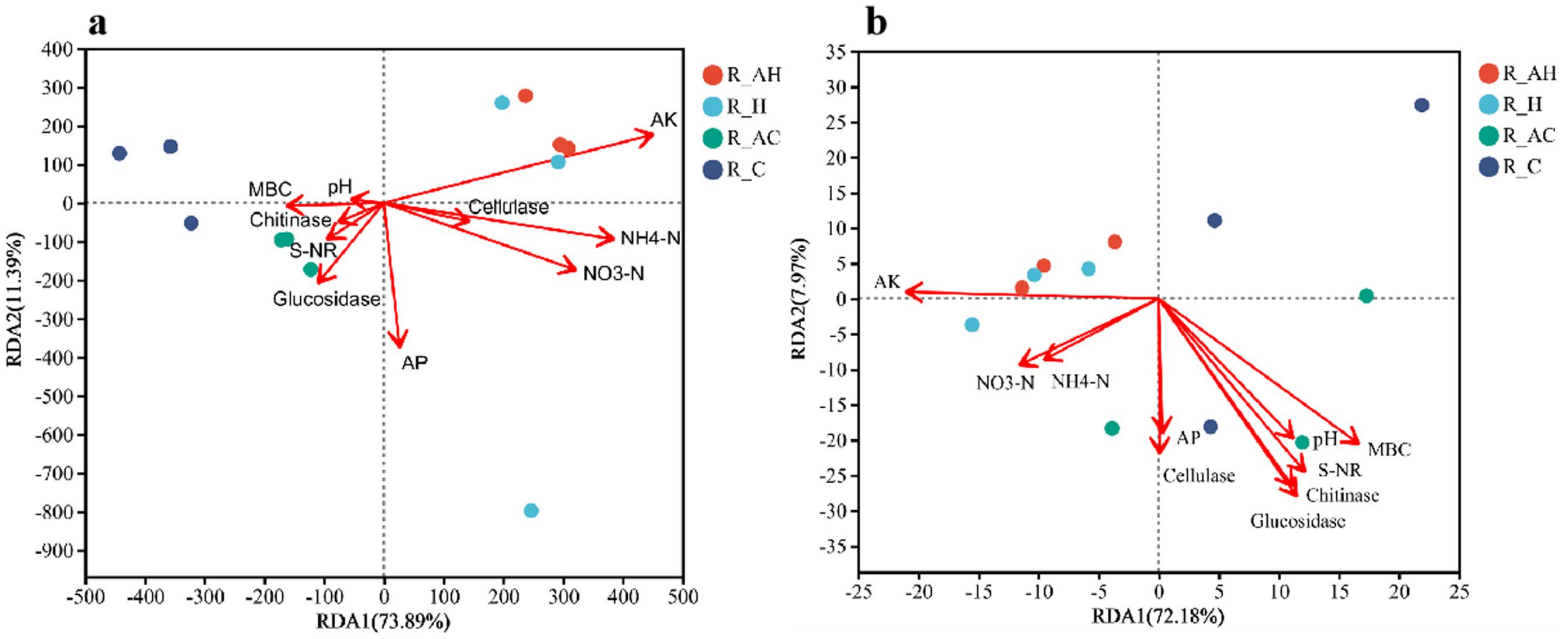
Redundant analysis (RDA) of the relationships between soil physicochemical properties and bacterial **(a)** and fungal **(b)** community composition under different treatments based on the genus level. AK, available potassium; NH_4_^+^-N, ammonium nitrogen; NO_3_^−^-N, nitrate nitrogen; AP, available phosphorus; pH, soil pH; MBC, microbial biomass carbon; Glucosidase, β-glucosidase activities; S-NR, nitrate reductase activities; Chitinase, chitinase activities; Cellulase, cellulase activities. R_AH, AMF-inoculated butterfly pea mycorrhizosphere soil, R_H, butterfly pea planting mycorrhizosphere soil, R_AC, AMF-inoculated control mycorrhizosphere soil, R_C, control mycorrhizosphere soil without AMF-inoculation.

We selected soil pH, available K, available P, NH_4_^+^-N, NO_3_^−^-N, MBC, cellulase, nitrate reductase, *β*-glucosidase, and chitinase as environmental factors to perform a RDA of soil bacteria and fungi across different treatments. In the mycorrhizosphere soil, available K (AK), NH_4_^+^-N, and NO_3_^−^-N were the primary factors influencing bacterial distribution (Figure 5a), whereas AK, MBC, S-NR, chitinase, and glucosidase had important effects on the fungal distribution (Figure 5b). The chemical factors explained 85.28 and 80.15% of the total eigen values in the bacterial and fungal RDA plots, respectively, indicating that physical and chemical factors had a relatively large impact on the content and distribution of microorganisms across the different treatments.

Based on the bacterial RDA plot (Figure 5a), the AH treatment affected AK, NH_4_^+^-N, NO_3_^−^-N, and cellulase in soil, whereas the AC treatment affected MBC, pH, cellulase, nitrate reductase, β-glucosidase, chitinase, and available P. In addition, based on the fungal RDA plot (Figure 5b), the AC treatment affected MBC, pH, nitrate reductase, chitinase, cellulase, β-glucosidase, and available P, whereas the AH treatment affected the soils’ NH_4_^+^-N, NO_3_^−^-N, and AK.

### 3.5 Spearman correlation between soil physicochemical properties and bacterial community

**Figure 6:**
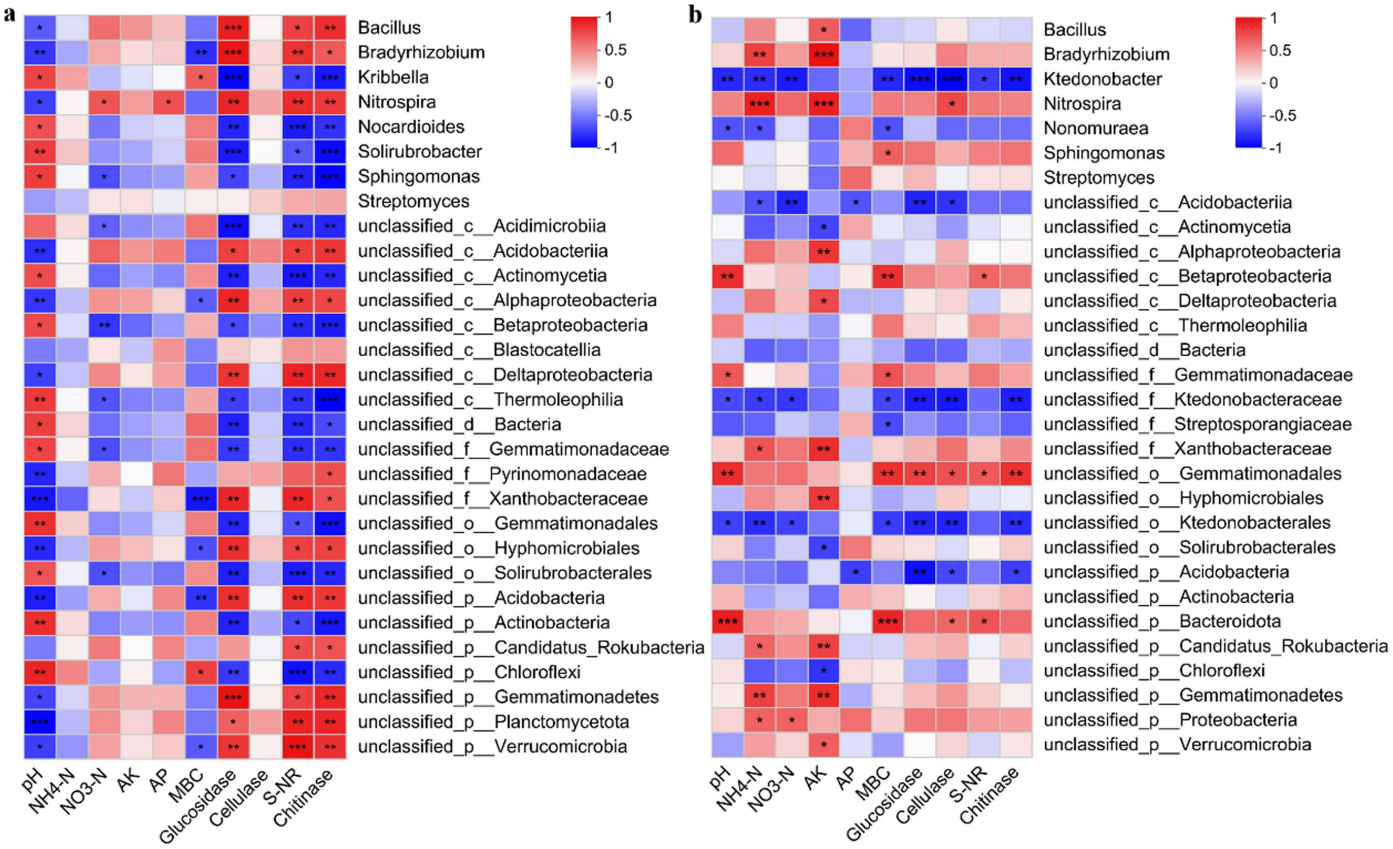
Spearman correlation between physicochemical properties and bacterial community composition in **(a)** bulk and **(b)** mycorrhizosphere soils under different treatments based on the genus level. Pearson’s correlation coefficient was used to evaluate the correlations. Blue denotes negative correlations, and red indicates positive correlations, with the color intensity reflecting the strength of the relationship. ^**^ *p* < 0.01, ^*^ *p* < 0.05.

A relationship between soil characteristics and specific microbial species was observed in both the bulk and mycorrhizosphere soils (Figures 6a,b). In bulk soil, pH was a key environmental variable affecting bacterial genera. In mycorrhizosphere soil, pH and MBC showed positive correlations with the bacterial genera *unclassified_c_Betaproteobacteria*, *unclassified_o_Gemmatiomadales*, and *unclassified_p_Bavteroidota*.

### 3.6 Soil metabolites in mycorrhizosphere

**Figure 7:**
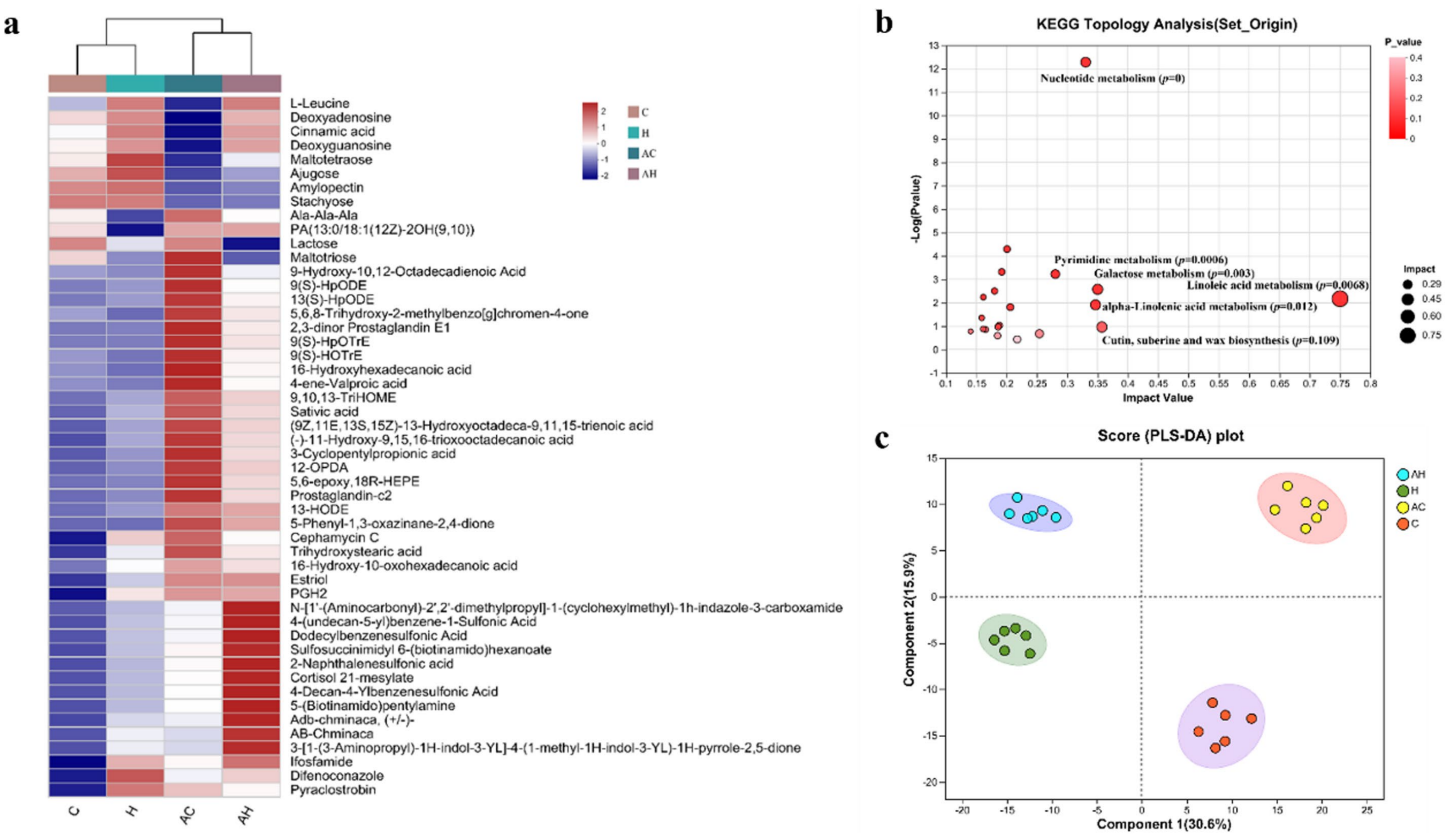
**(a)** Heat map, **(b)** metabolic pathway analysis, and **(c)** PLS-DA analysis of metabolites in the mycorrhizosphere soils (*n* = 6). The heat map displays sample names on the horizontal axis and metabolites on the vertical axis. Color variations indicate the relative abundance of metabolites, with red representing higher levels and blue representing lower levels. AH, AMF in butterfly pea planting soil; H, butterfly pea planting soil; AC, AMF with control soil (no planting butterfly pea soil); C, control soil (no planting butterfly pea soil).

The relative abundances of the 50 metabolites are presented as a heat map (Figure 7a). The detected metabolites include organic acids, amino acids, sugars, and several other substances. Relative to the AH, H, and C treatments, the AC treatment most strongly altered 9-Hydroxy-10,12-Octadecadienoic Acid, 13(S)-HpODE, 9,10,13-TriHOME, 13-HODE, 9(S)-HOTrE, 13-hydroxyoctadeca-9,11,15-trienoic acid, 9(S)-HpOTrE, 12-OPDA, maltotriose, and 16-hydroxy hexadecanoic acid. These metabolites were used to analyze the metabolic pathways (Figure 7b). Significant differences were observed in the metabolism of linoleic acid, nucleotides, pyrimidines, galactose, and alpha-linolenic acid (*p* < 0.05). The multivariate PLS-DA model was employed to derive the overall grouping information for the four treatment groups. The loading plot (Figure 7c) derived from the PLS-DA analysis revealed a clear distinction among the AH, H, AC, and C treatments.

**Figure 8:**
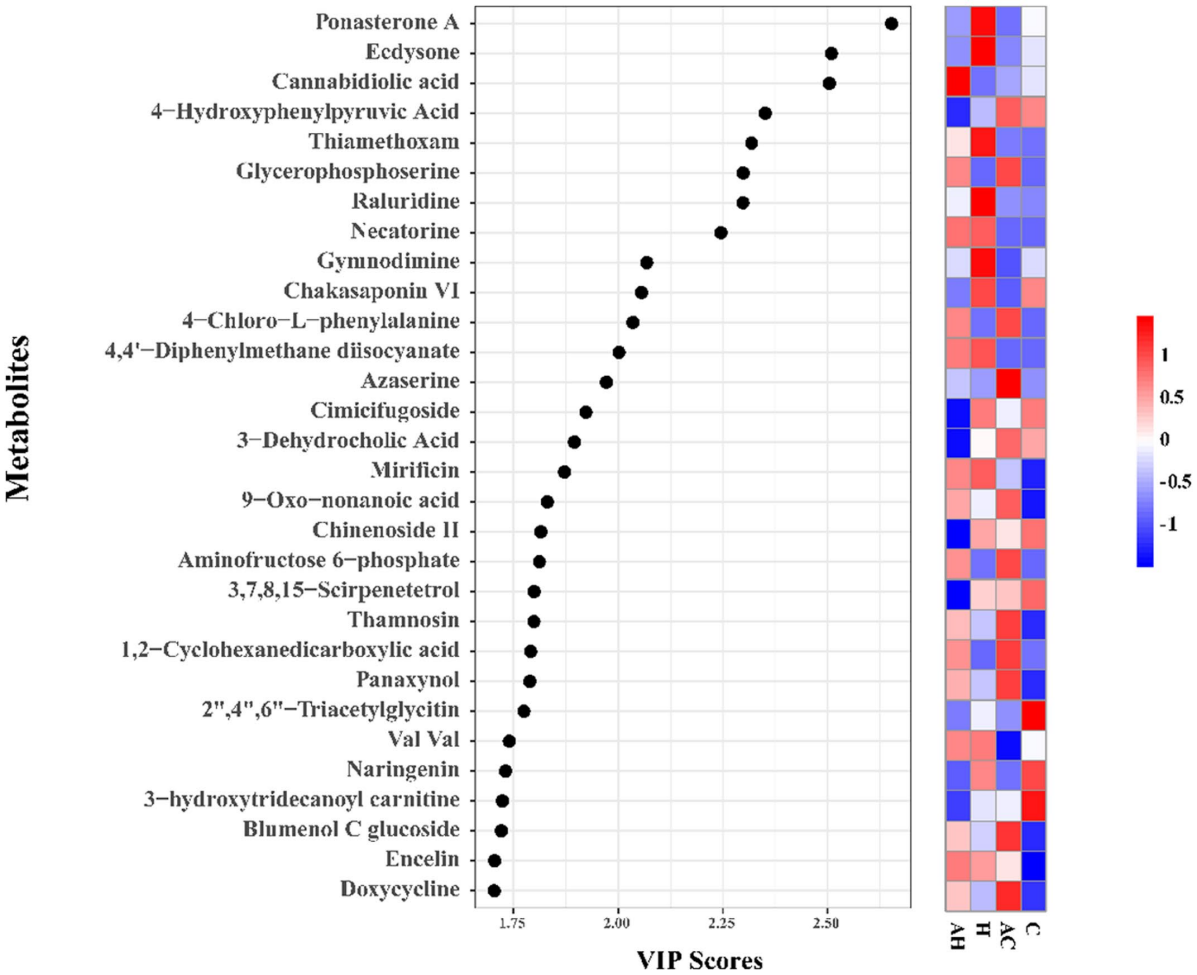
Variable importance in projection (VIP) scores of metabolites between AH, H, AC and C soils. AH, AMF in butterfly pea planting soil; H, butterfly pea planting soil; AC, AMF with control soil (no planting butterfly pea soil); C, control soil (no planting butterfly pea soil).

This distinct separation suggests that AMF and soil type substantially modified the low-molecular-weight metabolite profile in the soil. In the soil samples, 331 metabolites were identified using LC–MS-based non-targeted metabolomics. Subsequently, 28 metabolites, including cannabidiolic acid and hydroxyphenylpyruvic acid, were selected based on a variable importance in projection (VIP) score exceeding 1. These metabolites contributed to the divergent soil metabolomic profiles observed among the AH, H, AC, and C groups (Figure 8). In comparison to the C treatment, the AC treatment resulted in significantly higher levels of blumenol C glucoside, panaxynol, 1,2-cyclohexanedicarboxylic acid, thamnosin, aminofructose 6-phosphate, 9-oxo-nonanoic acid, azaserine, 4-chloro-L-phenylalanine, and glycerophosphoserine. However, compared to the H treatment, AH significantly increased cannabidiolic acid. This result indicates that AMF generally had a more pronounced and positive effect on nutrient-deficient soils.

### 3.7 Co-occurrence of environment variables, bacterial community, and metabolites in mycorrhizosphere soil

**Figure 9:**
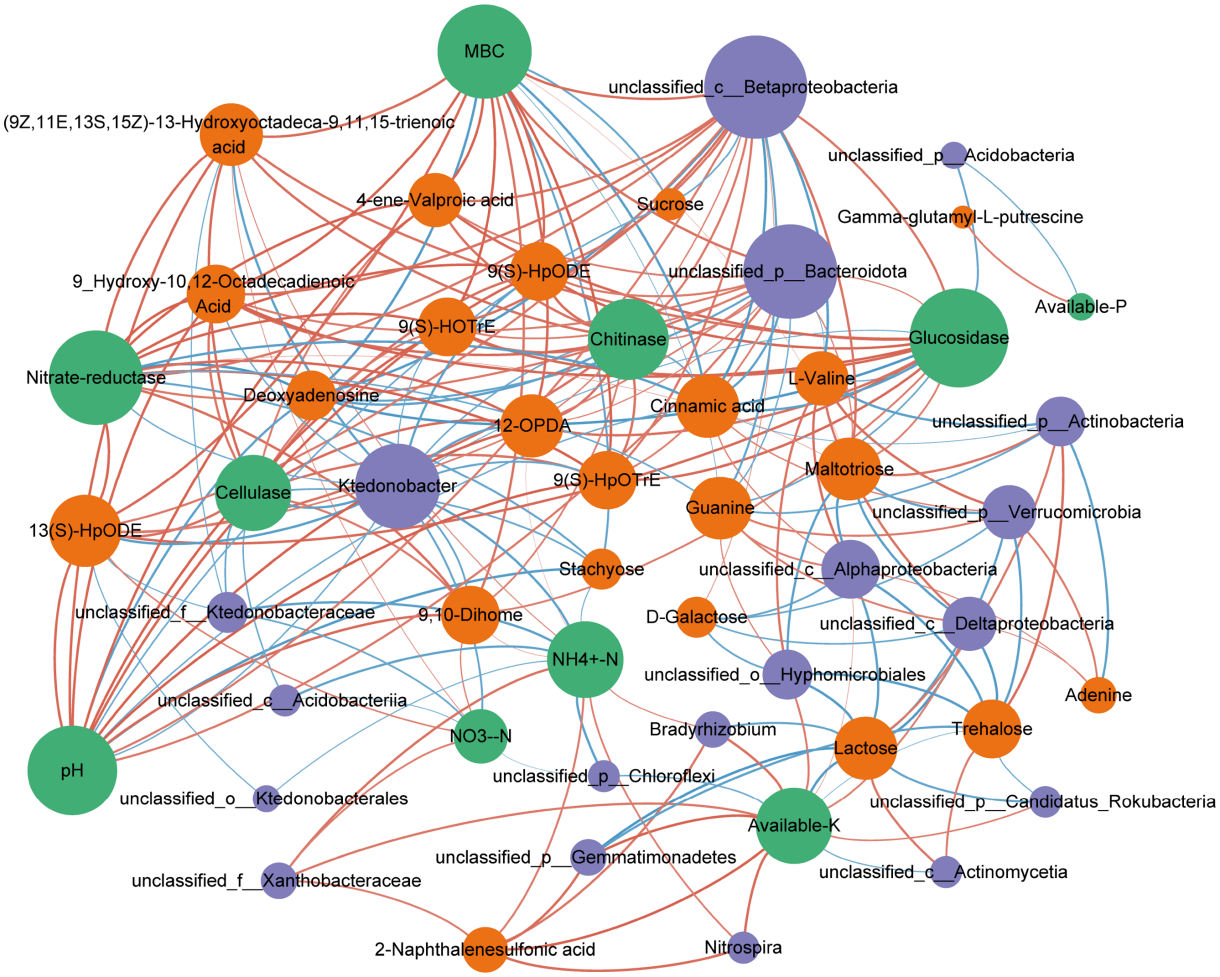
Co-occurrence analysis among soil bacterial taxa, soil properties, and soil metabolites in mycorrhizosphere soil. The node size is proportional to the number of connections (edges), and the edge width indicates the absolute value of the correlation.

We used a co-occurrence network to illustrate the interactions among the top 20 soil bacterial genera, soil physicochemical variables, and metabolites identified in our experiment (Figure 9). The network revealed a close distribution of bacterial communities, physicochemical variables, and metabolites within mycorrhizosphere soils. These findings indicate that soil metabolites crucially influence the composition of rhizospheric microbial communities. MBC, available K, nitrate reductase, glucosidase, and pH were more positively correlated with bacterial species. This finding suggests that these variables may stimulate most bacteria in the mycorrhizosphere soil.

MBC, nitrate reductase, glucosidase, pH, AK, and NH_4_^+^-N showed a greater number of positive correlations with metabolite species compared to negative correlations. This finding suggests that these variables may enhance the content of most metabolites, thereby influencing the soil bacterial community.

## 4 Discussion

### 4.1 Effects of AMFs and soil fertility on soil physicochemical characters and enzymes

The butterfly pea planted soil enhanced soil quality through increased accumulations of C and N in the soil (Chen et al., 2004). In our previous research, a five-year mango localization experiment showed that butterfly pea planted mango orchard soil had significantly higher NO_3_^−^-N, NH_4_^+^-N, AK, and AP contents compared to orchard soil without butterfly pea planted (Table 1). The results presented here are consistent with the earlier findings of Wang et al. (2016).

AMF-symbiotic plants significantly increased the pH of the mycorrhizosphere soil (Figure 1a), similar to the results reported by Kitayama (2013). In our experiment, the available P levels in the mycorrhizosphere soil across all four treatments were higher compared to those in the bulk soil. This is primarily attributed to the increased activity of soil enzymes in the mycorrhizosphere (Chen et al., 2020). Furthermore, the AMF exerted a more significant influence on the available P levels in the mycorrhizosphere soil (Figure 1e), indicating that the AMF enhanced the soil’s capacity to convert organic P into available forms. Xu et al. (2019) found that AMF increased the activity of phosphatase, which catalyzes the hydrolysis of organic P into inorganic P in soil. AMF markedly enhanced the MBC levels in both mycorrhizosphere and bulk soils compared to treatments that did not include AMFs (Figure 1b). MBC serves as a critical indicator of soil quality, thus reflecting the processes of nutrient transfer and energy cycling in the soil (Powlson et al., 1987). The AMF-induced increase in MBC was likely attributed to the enhanced growth and activity of microbes (Zhang et al., 2018; Kaiser et al., 2015). MBC represents a labile fraction of the soil organic carbon pool, and its concentration serves as a direct indicator of microbial biomass and metabolic activity. Furthermore, a significant positive correlation has been consistently observed between soil microbial abundance and MBC (Guan et al., 2022). A more significant effect of AMF on microbial diversity was observed in low-fertility soils relative to high-fertility soils. These conditions also enhance the decomposition and absorption of resources, thereby supplying essential nutrients to host plants (Zeng et al., 2020; Chen et al., 2020). Therefore, low-fertility soil (AC) had a significantly higher MBC concentration than high-fertility soil (AH) in the present study. During evolution, mycorrhizal plants have developed dual nutrient acquisition pathways: (1) the direct pathway through root hairs and epidermal cells, and (2) the indirect mycorrhizal pathway via AMF (Smith and Smith, 2011). The growth and nutrient transport activities of AMF are energy-demanding processes. While the mycorrhizal pathway enables plants to acquire nutrients at extremely low concentrations through expanded absorption surface area, the root pathway becomes more cost-effective when nutrient availability is high (Chen et al., 2017). Consequently, AMF play a diminished role in high-fertility soils. In our experiment, AMF increased NH_4_^+^-N and NO_3_^−^-N concentrations (Figures 1c,d). Previous research has also demonstrated that AMF can promote N mineralization and consequently influence plant growth (Li et al., 2013).

In nutrient-deficient soils, host plants are more likely to depend on mycorrhizal associations for nutrient acquisition (Smith and Smith, 2011). The interaction between mycorrhizae and microorganisms can stimulate microbial activity, leading to increased secretion of enzymes that aid in the mineralization and decomposition of organic matter and thereby enhancing nutrient cycling (Zhou et al., 2020; Phillips et al., 2011). In our study, the butterfly pea planted soil had higher soil nutrient contents than the control soil (Table 1). The soil enzyme activity rates of plants that were not inoculated with AMF were higher in soil H than in soil C. However, in the AMF-inoculated treatment, soil enzyme activity rates were higher in AC soil than in AH soil, particularly in the mycorrhizosphere soil (Figures 2a–d). These findings align with those of previous research demonstrating that soil enzyme activities in nutrient-poor soils are higher in AMF-inoculated treatments than in nutrient-rich soils (Chen et al., 2020). Soil enzymes, including cellulase, *β*-glucosidase, and chitinase, strongly influence the carbon cycle (Kohler et al., 2015). AMF significantly affected cellulase, β-glucosidase, and chitinase activities in the mycorrhizosphere soil (Figures 2a,c,d). Various studies have highlighted a robust positive association between soil MBC and enzyme activity (Zhang et al., 2020; Fall et al., 2016), a finding that aligns with our experimental results. Several previous studies indicated that AMF can produce exudates, which are beneficial for promoting microbes in the soil. These microbes can then enhance the activity rates of cellulase, β-glucosidase, and chitinase, increasing soil nutrient levels (Caravaca et al., 2004; Johansson et al., 2004; Kohler et al., 2015). In our experiment, the AC treatment had the highest nitrate reductase activity in the mycorrhizosphere soil, followed by the AH treatment (Figure 2b). Nitrate reductase plays a crucial role in reducing NO_3_^−^-N to NH_4_^+^-N in soil. The existence of AMF led to improved organic matter decomposition via enhanced microbial biomass and increased nitrate reductase activity (Kong et al., 2018).

### 4.2 Effects of AMFs and soil fertility on bacteria and fungi

Utilizing high-throughput sequencing techniques, we analyzed the bacterial and fungal communities present in both bulk soil and mycorrhizosphere soil. In the latter soil, both the Chao and Simpson indices were significantly higher in the AC treatment than in the C treatment. This finding implies that AMF positively influence the richness and diversity of soil microbial communities (Table 4). AMF promote the growth and activity of free-living microorganisms via exudates released from their hyphae (Toljander et al., 2007; Kaiser et al., 2015). In the present study, the AC treatment had significantly higher Chao and Simpson indices than the AH treatment in the mycorrhizosphere soil. This result may have occurred due to limited nutrient resources in low-fertility soil (AC) environments, which can inhibit plant growth and increase the dependence of plant productivity on AMF (Supplementary Table S1) (Chen et al., 2020). In high-fertility soils (AH), however, limited carbon was allocated to microorganisms, which caused plants to absorb more nutrients through their roots (Zeng et al., 2020; Janssens et al., 2010; Li et al., 2015).

The composition of microbial communities is intimately linked to soil quality and plant health (Berendsen et al., 2012). Numerous researchers have found that AMF can recruit several types of beneficial bacteria into the soil, thereby altering the ecological environment (Xu et al., 2019). Our experiment corroborated these findings. In the bulk soil, the relative abundances of *unclassified_p_Actinobacteria* increased in the AH and AC treatments compared to the H and C treatments (Figure 3). *Actinobacteria* are important plant-associated microorganisms that enhance resistance to both biotic and abiotic stresses (Palaniyandi et al., 2013; Qu et al., 2021). *Unclassified_p_Chloroflexi* significantly impacts the maintenance of soil organic matter and nutrient cycling due to its unique photosynthetic capabilities (Qu et al., 2021). Within the mycorrhizosphere soil, the relative abundance of this group was more pronounced in the AC treatment in comparison to the remaining treatments. Overall, these results indicate that AMF recruited potentially beneficial microorganisms that influenced soil nutrients. The interaction between bacteria and AMF facilitates a crucial and efficient pathway for plant nutrient acquisition from organic materials (Bonfante and Anca, 2009). Furthermore, the extraradical mycelia of AMF offer the essential physical infrastructure that supports microbial colonies, enabling them to capture and decompose organic matter (Gahan and Schmalenberger, 2015; Jiang et al., 2021). The relative proportions of *unclassified_p_Chloroflexi* and *unclassified_c_Actinomycetia* in the AC and C treatments in the bulk soil increased compared to those in AH and H, indicating that the two bacteria and high-fertility soils had a close relationship.

The impact of AMF on fungal diversity and abundance in the soil was relatively smaller than its effect on bacteria. The AH and AC resulted in a higher relative abundance of *Rhizophagus* in both bulk and mycorrhizosphere soils (Figures 3c,d). The relative abundances of *Aspergillus*, *Rhizopus*, *Claroideoglomus*, *Gigaspora*, and *Ambispora* in the AH, H, AC, and C treatments were similar in both the bulk and mycorrhizosphere soils. The presence of *Claroideoglomus*, *Gigaspora*, and *Ambispora* in all four treatments may have resulted from the unsterilized experimental soil. Jiang et al. (2020) investigated AMF resources in the mycorrhizosphere soil of mango orchards in southern China and identified *Diversispora*, *Claroideoglomus*, *Gigaspora*, and *Ambispora*. This finding suggests that *Claroideoglomus*, *Gigaspora*, and *Ambispora* are native AMF species of mangos, though their abundances were not high. These results indicate that soil nutrients significantly influenced the bacterial community structures in both bulk and mycorrhizosphere regions.

In our study, considering the results of PCoA (Figure 4) and RDA (Figure 5), the AH and AC treatments altered the bulk and mycorrhizosphere bacterial community structures. The correlation analysis between bacterial communities and physicochemical properties of the mycorrhizosphere soil revealed that NH_4_^+^-N was positively associated with the abundances of *Bradyrhizobium* and *Nitrospira* (Figures 6a,b). *Bradyrhizobium* ensures an adequate supply of N to plants by fixing N from the soil air (Wang et al., 2020). *Nitrospira* is the main nitrite-oxidizing bacterium influencing nitrification; its absence disrupts nitrate and nitrite circulation systems, resulting in soil nutrient and fertilizer losses (Qu et al., 2021). AK was positively correlated with *unclassified_f_Xanthobacteraceae*, which promotes plant growth in several ways, including activating plant hormones and dissolving minerals. pH, MBC, glucosidase, cellulase, S-NR, and chitinase were positively correlated with *unclassified_o_Gemmatimonadales,* which participates in ecological processes such as organic matter decomposition (Liu et al., 2016).

### 4.3 Effects of AMFs and soil nutrients on metabolites

Soil metabolites primarily originate from the decomposition of plants, root exudates, soil organic matter, microbes, and microbial metabolites (Cheng et al., 2018). Modifications in the composition and levels of soil metabolites can reveal information about the present and historical responses of soil microorganisms to nutrient environments (Li et al., 2019). The present results showed that the metabolites in the mycorrhizosphere soil were altered by AMF and the butterfly pea cover (Figure 7a). A total of 331 metabolites, including lipids, organic acids, amino acids, and sugars, were identified in the soil samples. These metabolites, primarily organic acids, function as key metabolites and significantly influence the composition of soil microbial communities in the mycorrhizosphere (Shi et al., 2011). The composition of microbial communities near plant roots is strongly influenced by organic acids (Badri et al., 2013), which also provide essential carbon resources for the proliferation of bacteria (Qu et al., 2021). The increase in organic acid levels in the mycorrhizosphere of low-fertility soil inoculated with AMF (AC treatment) is likely highly influential in converting soil-insoluble P compounds into an available form, thereby enhancing P uptake by plants (Li et al., 2007).

The metabolic pathway that underwent the most significant changes in the mycorrhizosphere soil was the linoleic acid metabolism (Figure 7b). Furthermore, several pathways associated with carbon metabolism, including pyrimidine metabolism, galactose metabolism, and alpha-linolenic acid metabolism, were substantially impacted (Zhang et al., 2020). Prior research has shown that soil metabolomics offers valuable insights into mycorrhizosphere carbon fluxes at the molecular level (Song et al., 2020). As illustrated in the PLS-DA score plot (Figure 7c), the AH, H, AC, and C treatments were separated, indicating that AMF and nutrient-rich soil significantly affected the mycorrhizosphere soil metabolic profiles. Soil microorganisms contribute substantially to the overall soil metabolite pool through the release of both intracellular and extracellular metabolites (Zhang et al., 2020).

The VIP score is a critical measure for identifying key predictors in PLS-DA (Figure 8). Based on the VIP > 1.5 parameters, important variations in 30 metabolites were identified in the mycorrhizosphere soils. The pathway enrichment analysis revealed specific changes in soil metabolic processes. In the low-fertility soil with the AMF treatment (AC), metabolites such as glycerophosphoserine, 4-chloro-L-phenylalanine, azaserine, aminofructose 6-phosphate, and blumenol carbon glucoside were significantly enriched in the mycorrhizosphere, suggesting potential roles in promoting plant growth. Amino acids in soil significantly influence soil fertility and plant productivity by regulating the dynamic metabolism of carbon and N in soil (Moreira and Moraes, 2016). Furthermore, in low-fertility soil treated with AMF (AC treatment), amino acids and sugars serve as key carbon sources for mycorrhizosphere microorganisms, thus positively influencing plant growth and the soil microenvironment (Zeng et al., 2020). Metabolites associated with amino acid metabolism and carbon fixation pathways exhibited significant correlations with AMF functionality (Zhang et al., 2024). Overall, our research findings align with previous studies, confirming that AMF can enhance the chemical diversity of mycorrhizosphere metabolites and augmenting specific components.

### 4.4 Effects of AMFs and soil nutrients on the correlation of microbes and metabolites

The co-occurrence network results provide a new perspective for analyzing interactions among soil properties, bacterial taxa, and soil metabolites (Ziegler et al., 2018). In our experiment, correlation-based network results (Spearman’s r > 0.7 or r < −0.7, and *p*-value <0.05) revealed significant associations among soil properties, bacterial taxa, and soil metabolites (Figure 9). A considerable body of research has indicated that soil metabolites are essential in determining the composition and functionality of microbial communities in soil (Li et al., 2022; Shimasaki et al., 2021). Alterations in soil metabolites influence the composition, distribution, and abundance of soil microorganisms (Fu et al., 2022) and can substantially influence soil properties (Cai et al., 2021). A substantial amount of research has shown that alterations in soil metabolites can recruit diverse beneficial microbial communities, thereby enhancing nutrient availability in the soil and subsequently promoting plant nutrient uptake (Wen et al., 2022; Yuan et al., 2018). Soil metabolites have diverse effects on soil microbiomes as signaling molecules, including promotion, inhibition, and eviction (Yue et al., 2023). Similarly, the metabolic activities of soil microbiomes are highly influential in shaping soil metabolite composition (Cheng et al., 2022). A close association between bacterial activity and soil metabolites has been previously reported (Ding et al., 2021). The external addition of AMF had a notable effect on increasing the pH levels and MBC in the mycorrhizosphere soil. These increases modulated soil metabolites such as 9(S)-HpOTrE, 9(S)-HOTrE, 12-OPDA, 4-ene-valproic acid, 9(S)-HpODE, and 9-hydroxy-10,12-octadecadienoic acid, which in turn increased the abundances of key bacteria such as *unclassified_c_Betaproteobacteria* and *unclassified_p_Bacteroidota* (phylum Bacteroidota). Metabolites 9(S)-HpOTrE, 9(S)-HOTrE, 12-OPDA, 4-ene-valproic acid, 9(S)-HpODE, and 9-hydroxy-10,12-octadecadienoic acid are classified as fatty acid derivatives. Song et al. (2020) demonstrated that their involvement in carbon metabolic pathways, where these metabolites serve as vital carbon and energy sources for soil microbial communities. *Unclassified_c_Betaproteobacteria* was identified as the dominant bacterial genus. This taxonomic group includes rhizobial species known to participate in N cycling through biological N fixation (Chen et al., 2022). Our study supports the significant association among soil metabolites, bacteria, and nutrients. Xu et al. (2019) also found that interactions between bacteria and their metabolites were important for soil nutrient cycling. In conclusion, the close relationship among soil metabolites, bacteria, and nutrients suggests mutual modulation of root activities by plants and soil bacteria through metabolic processes, ultimately affecting crop productivity.

## 5 Conclusion

This study explored how externally introduced AMF affect the cycling of nutrients in soil. The exogenous addition of AMF under low-fertility conditions (AC) markedly altered the diversity of soil bacterial communities, metabolite profiles, and metabolic pathways in the mycorrhizosphere soil of mangos. The structure of the bacterial community was strongly correlated with metabolite profiles. The AC treatment recruited potentially beneficial mycorrhizosphere microorganisms and resulted in higher levels of metabolites, such as organic acids. The addition of AMF affected soil pH, MBC, NH_4_^+^-N, NO_3_^−^N, AK, and AP, which reshaped the soil bacterial community and altered the metabolite composition. However, the effect of AMF on soil nutrient availability was diminished in high-fertility soils. The results of this study may contribute to a deeper comprehension of nutrient transformation in high-and low-fertility soil ecosystems using exogenous AMF and provide a reference for nutrient utilization at varying soil nutrient levels.

## Data Availability

Sequencing was performed on the Illumina PE300/PE250 platform, and the raw sequencing data were deposited in the NCBI SRA database under accession number PRJNA273116.
