# Supplementary material for: Arbuscular mycorrhizal fungi enhance soil nutrient cycling by regulating soil bacterial community structures in mango orchards with different soil fertility rates
Source: Front Microbiol. 2025 Jun 27;16:1615694. doi: 10.3389/fmicb.2025.1615694 (PMC12245812; doi:10.3389/fmicb.2025.1615694)
Supplement: Supplementary file 1 [file Data_Sheet_1.docx]

**Supplementary material**


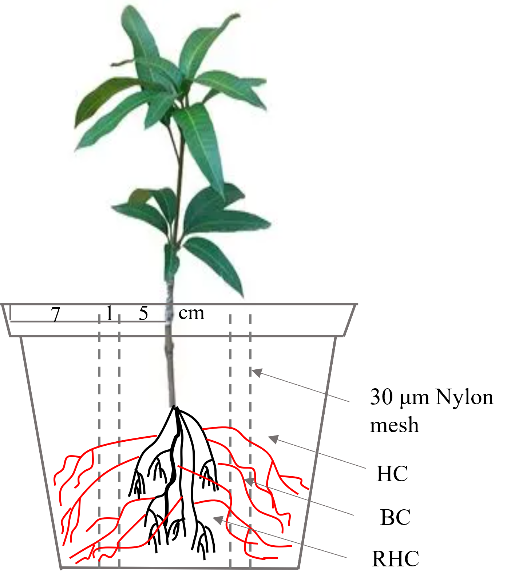


**Figure S1** Schematic diagram of the three-compartment experimental set-up. The inner compartment contains the roots and fungal hyphae (RHC), the middle compartment is buffer compartment (BC) and the outer compartments is the hyphal compartment (HC) only containing the extraradical hyphae.
