# Supplementary material for: Arbuscular mycorrhizal fungi enhance soil nutrient cycling by regulating soil bacterial community structures in mango orchards with different soil fertility rates
Source: Front Microbiol. 2025 Jun 27;16:1615694. doi: 10.3389/fmicb.2025.1615694 (PMC12245812; doi:10.3389/fmicb.2025.1615694)
Supplement: Supplementary file 2 [file Data_Sheet_2.docx]

**Supplementary material**

**Table S1** Changes in mycorrhizal colonization in the different treatments.

| Treatments | Colonization (%) |
| --- | --- |
| AH | 47.67±2.52 b |
| H | 8.03±0.25 c |
| AC | 60.33±4.51 a |
| C | 8.9±0.36 c |

AH: AMF in butterfly pea planting soil, H: butterfly pea planting soil, AC: AMF with control soil (no planting butterfly pea soil), C: control soil (no planting butterfly pea soil). Data are presented as means ± SE (n = 3), where different letters within columns denote significant differences (*P* < 0.05).
